# Flipped Presentation of Authentic Audio-Visual Materials: Impacts on Intercultural Sensitivity and Intercultural Effectiveness in an EFL Context

**DOI:** 10.3389/fpsyg.2022.832862

**Published:** 2022-02-21

**Authors:** Masoud Khabir, Ali Akbar Jabbari, Mohammad Hasan Razmi

**Affiliations:** Department of English Language and Literature, Yazd University, Yazd, Iran

**Keywords:** flipped classroom, intercultural sensitivity, intercultural effectiveness, authentic materials, EFL context, learners' perceptions

## Abstract

Utilizing a pre-experimental pre-test post-test design, this study investigated the effect of an authentic audio-visual American sitcom (*Friends*) on the intercultural sensitivity (ICS) and intercultural effectiveness (ICE) of a sample of male and female upper-intermediate English students. To this aim, 34 Iranian EFL students were selected through convenient non-random sampling. In order to assure the participants' homogeneity in English proficiency, the selected students were given the Oxford Quick Placement Test prior to the intervention. Over a 10-week period, the participants were presented with nearly 30-min-long episodes of *Friends* in a flipped context, two episodes every week uploaded to the accompanying website. An online 10-item quiz on the day of watching and a next-day meeting were held. During the online discussion meetings, the participants discussed cultural questions tailored to the aims of the study. The required data were collected through the administration of the intercultural sensitivity scale (ISS) and intercultural effectiveness scale (IES) in pre- and post-test assessments. The analyses of the data indicated that both ICS and ICE improved significantly during the intervention. However, ICS indicated more improvement. In addition, a semi-structured interview was administered to the participants to examine their perception of the flipped classroom experience. Having analyzed the data using MAXQDA 2020, some significant themes emerged which are reported. On the whole, the participants portrayed positive attitudes toward using technology in flipped classes. The educational and practical implications are discussed.

## Introduction

Since culture and language are closely entwined, it seems problematic to teach language without cultural conceptualizations (Byram and Feng, 2004; Lantolf, 2006; Risager, 2007, 2020; Thorne and Lantolf, 2007; Baker, 2012; Sharifian, 2012; McConachy, 2017). Lave and Wenger (1991, p. 73) well contends that “language and culture should be seen as a whole” and underlain in languaculture, which reflects Vygotskian (1978) theory, i.e., learning is initially social and then individual (Agar, 1994; Risager, 2012). This relationship is complicated due to the fact that the goals of learning foreign languages have recently changed from linguistic to intercultural communication (Byram, 1997; Sercu, 2005; Piasecka, 2011), and cultural systems may be associated with one or more than one language (Sharifian, 2012). Similarly, non-native English speakers must deal with their native language culture, foreign language culture, and interlocutors' culture.

It has been appropriately argued that communicating in English with individuals from different cultures is an important ELT topic (Nieto and Zoller Booth, 2010; Baker, 2012; Widodo et al., 2017; Tofighi et al., 2021). Foreign language learners (EFLers), in this regard, need to be exposed to a diverse range of authentic cultural representations to develop an awareness of the intended language–culture (Byram, 1989; Lave and Wenger, 1991; Roberts et al., 2000; Richards, 2005; Beresova, 2015; Akbari and Razavi, 2016; Fernández-Agüero and Chancay-Cedeño, 2018). The benefits of authentic materials include enhancing comprehension, providing specific language, adding cultural points, boosting motivation, and broadening knowledge of the language (Kim, 2015; Badri and Salehi, 2017). Furthermore, authentic materials encourage EFLers to notice, respect, and appreciate cultural variations (Davis, 1997; Smith and Rawley, 1997; Erkaya, 2005).

Additionally, authentic audio-visual materials facilitate the development of foreign language skills by integrating image, sound, and culture (Baltova, 1999; Vanderplank, 2010). Equally, Desai et al. (2018, p. 405) hold the view that “In cases where actual exposure to other cultures is not feasible, films can be a medium of simulation to immerse oneself in a different culture.” Desai et al. (2018) believe that since films represent culture visually, they will help students reconsider their current beliefs about other cultures, explore peculiarities, accept, and eventually adapt to them. Accordingly, sitcoms, such as *Friends*, in which one can clearly observe how people interact and behave during events like Thanksgiving and Christmas (Mudawe and Mudawe, 2020), are appropriate resources for integrating language and culture (Kozhevnikova, 2014).

On the other hand, research has shown that the accessibility, along with the convenience and ubiquity of technology-mediated instruction, fosters self-regulated learning, which is a key concept in flipped classrooms (FCs; Clark, 2007; Murdock and Williams, 2011; Chen Hsieh et al., 2017; Namaziandost et al., 2021). FC effectively integrates tradition and online education by exploiting both in- and out-of-class time (Mattis, 2015), through presenting pre-recorded video clips prior to the class, allowing the students to learn at their favorable pace, pause, rewind, and replay the videos (Yu and Wang, 2016; Chen Hsieh et al., 2017; Tsai, 2019), leading to a more active type of learning (Galway et al., 2015). According to Doman and Webb (2017), online activities help shy and apprehensive-of-speaking-in-class students more. To fulfill this aim, using communication technology for presenting pre-recorded videos is essential (Yu and Wang, 2016; Chen Hsieh et al., 2017).

Due to the inter-relationship between culture and language learning being ignored in an EFL context like Iran (Turizo and Gómez, 2006; Sharifian, 2012; Ajabshir, 2019; Razmi et al., 2020; Tirnaz and Haddad Narafshan, 2020), and the impracticality of playing videos in classroom considering the facilities and timing, FC seems an appropriate choice for both incorporating culture and presenting it.

Furthermore, an important factor that predicts the students' motivation in learning is the positive perception of the learning environment (Unsal, 2012). Ryan and Deci (2000) claim that if students are free to self-regulate their learning in electronic environments, their intrinsic motivation will be encouraged. Feelings of competence will enhance intrinsic motivation when accompanied by a sense of autonomy (Ryan and Deci, 2000). Out-of-class activities will intrinsically motivate students more than the traditional mode of practicing activities (Abeysekera and Dawson, 2015). By providing flexible learning environments (Yarbro et al., 2014), out-of-class exercises, and improving students intrinsic motivation (Johnson, 2013; Chen Hsieh et al., 2017; Thai et al., 2017), FC has the potential to attract promising perceptions (González-Gómez et al., 2016; Adnan, 2017; Awidi and Paynter, 2019). Accordingly, knowing about students' perception, which is reflective of their motivation, seems significant.

This study, therefore, sought to fill the culture–language gap by using the American *Friends* sitcom as a culturally rich resource (Kozhevnikova, 2014; Mudawe and Mudawe, 2020) in a way that students may be more sensitive and responsive to cultural diversities and hence gain proficiency in their English learning undertaking. Moreover, the students' perceptions toward the application of technology in a flipped setting is another objective of the present investigation. In what follows, the theoretical framework of the study and the research questions are presented.

## Theoretical Framework

The present study draws on cultural-learning frameworks in presenting audiovisual materials and devising discussion questions. Considering in-class activities, concepts of community of practice (Vygotsky, 1978), situated learning, legitimate peripheral participation and learning, the importance of social interactions preceding learning development (Lave and Wenger, 1991), and scaffolding apprentices, more knowledgeable other (MKO), zone of proximal development (ZPD), collaborative learning, and reciprocal teaching-learning (Vygotsky, 1978b) were carefully considered.

Deardorff's (2006) process model of effective and appropriate ongoing development of intercultural competence in intercultural situations proposes a developmental cycle of individual's attitude (respect, openness, curiosity, and discovery), global knowledge and skill (observation, listening, evaluating, analyzing, interpreting, and relating), and internal (empathy), and external outcomes (effectiveness and appropriateness). Deardorff's (2004) intercultural pyramid model shares a lot with his process model; the former is linear but the latter is cyclic, where individuals can enter at any stage. However, the degree of their achievement may differ.

Furthermore, comparing Deardorff's (2006) process model with Byram's (1997) five *Savoirs*, it seems that an individual's attitude, knowledge, internal outcomes, and external outcomes correspond to *Savoir être, Savoir, Savoir comprendre, Savoir apprendre/faire*, and *Savoir s'engager*, respectively. In addition, Bennett and Bennett's (2004) proposed spectrum containing seven instances of denial, defense, reversal, minimization, acceptance, adaptation, and integration has a lot in common with Deardorff's and Byram's models.

Drawing on the aforementioned theoretical grounds, the pre-viewing, while-viewing, and post-viewing stages of this research draw on the process model of Deardorff. The strategies and questions utilized build on Vygotsky's (1978a), Deardorff's (2006), Bennett and Bennett's (2004), and Lave and Wenger's (1991) beliefs as their theoretical foundations. In line with Vygotsky, maintaining that individual development cannot be understood without reference to the social context, this research used the *Friends* sitcom as the authentic sociocultural context containing authentic cultural instances (e.g., Thanksgiving and Christmas).

Following the illustration of Yarbro et al. (2014), the researchers designed FC instruction with four major pillars of FC: flexible environment, learning culture, intentional content, and professional educator. The pillars of FC were met through the utilization of online presentations, devoting class time to discussing cultural matters, selected *Friends* episodes by a CELTA-certified teacher-researcher.

## Review of Literature

This section pays special attention to the literature significant to our research. Unlike traditional teacher-centered classrooms where students were treated as *table blanche* absorbing information passively (Betihavas et al., 2016), FC is student-centered (Bergmann and Sams, 2012). The rationale for using FC is its strength in increasing student–student and student–teacher interactions (Lage et al., 2000; Thorne et al., 2009; Murdock and Williams, 2011; Bergmann and Sams, 2012; Boucher et al., 2013; Enfield, 2013; Schultz et al., 2014), creating more diverse in- and out-of-class learning materials and activities (Gannod et al., 2008; Bishop and Verleger, 2013), helping learners become more empowered to take responsibility for their own learning, and thereby promoting self-regulated learning (Laman et al., 2012; Mok, 2014). Students in this model have greater opportunities to become autonomously reflective learners (Enfield, 2013).

Akçayir and Akçayir's (2018) review of 71 related research articles revealed that the improvement of students' learning performance was the most frequently reported advantage of the FC. The theoretical foundations of the FC advocate the view that class time should not be merely devoted to delivering one-sided lectures (Bishop and Verleger, 2013).

Additionally, Tsai (2019) mentioned that learning diversity can be reinforced by FC, enabling students to learn over various channels using electronic materials, merely by pausing or rewinding the audio-visual materials. Though, some authors contend that lectures, being pre-recorded, are similarly effective for learning as in-person lectures (McNeil and Nelson, 1991; Zhang et al., 2006). Mok (2014) confirms the benefit of FC by mentioning that students could watch videos many times before the class. Some research found out that by using digital and interactive platforms, FCs can provide distinguished instruction and empower the learning process adapting to the learners' learning styles and pace (Bull et al., 2012; Bishop and Verleger, 2013; Enfield, 2013). Johnson and Marsh (2014) and Tsai (2019) also report that the students become more autonomous in the FC model of self-regulated learning.

Johnson and Marsh (2014) and Mehring (2016) revealed the potential of FCs in EFL education through forming a cooperative communicative language learning environment incorporating language reinforcement activities. Wu et al. (2017) indicated that students' oral proficiency on the IELTS assessment criteria improved significantly after FC instruction.

Some other researchers found that the administration of FC fostered opportunities for learners to structure their knowledge, deliver their ideas, and then experiment with their interpersonal communication skills (Arnold-Garza, 2014; Butt, 2014; Adnan, 2017).

The promising impact of FC on academic settings has been well-reported in many different subjects (Deslauriers and Wieman, 2011; Baepler et al., 2014; Schultz et al., 2014), including foreign language education (Turan and Akdag-Cimen, 2020). Yu and Wang (2016) indicated an improved performance of students undergoing FCs in English business writing.

Tofighi et al. (2021) contend that it is necessary to incorporate ICC in classes. In a study, Desai et al. (2018) investigated the effect of flipped presentation of culture through popular feature films on the cultural intelligence of participants and concluded that FC increased students' cultural intelligence levels. Zhang (2020) explored the development of Chinese learners' intercultural competence through authentic video and found out that their intercultural competence expanded, and students became more culture-tolerant, empathetic, and sensitive. In another study, Jensen (2019) examined the effect of FC on L2 students' cross-cultural critical thinking and found that students' cross-cultural critical awareness improved.

The findings of Zainuddin and Attaran (2016) depicted that not only did students possess positive attitudes toward FCs but also they recommended FCs for other courses and students. This is due to the fact that students believe FC catalyzes learning. Research also indicates that shy and quiet as well as full-time international students are positively impacted by FC, though part-time students face challenges (Zainuddin and Attaran, 2016). The post-survey data by Galway et al. (2015) indicated positive perceptions of students toward FC. Another study (He et al., 2016) found that students had contrasting opinions toward FC; however, their positive attitudes were not significantly different from the neutral position.

The studies done so far have focused on the effect of FC on learners' intercultural communicative competence in an ESL context. What is not yet clear is the examination of the topic in an EFL context where learners have little or no access to intercultural communication in a foreign language. This study is, therefore, an attempt in this regard. Utilizing a pre-experimental pre-test post-test design, the study attempts to investigate the effect of an authentic audio-visual American sitcom (*Friends*) on Iranian EFL learners' intercultural sensitivity (ICS) and intercultural effectiveness (ICE). Moreover, the students' perceptions toward the implementation of FC are probed. Specifically, this investigation addresses the following research questions:

**RQ1**. Does the implementation of flipped classroom have an impact on students' intercultural sensitivity?
**RQ2**. Does the implementation of flipped classroom have an impact on students' intercultural effectiveness?
**RQ3**. What is the effect of the implementation of flipped classrooms on the learners' perception?

## Method

### Research Design

This study is a pre-experimental pre-test post-test design. A single case of 34 students was observed at two time points, one before the treatment and one afterward. Changes in the outcome were presumed to be the result of the intervention. No control or comparison group was employed. This research was a part of a wider study containing qualitative sections, which could not be reported here due to the word count limitations.

### Participants

This study was conducted with upper-intermediate female and male students in Kerman institutes. The participants were selected through convenience non-random sampling. To ensure the homogeneity of the participants in terms of the level of L2 proficiency, the Oxford Quick Placement Test (OQPT) was conducted. The initial number of participants was 43. However, analyzing the OQPT results, 5 learners were discarded from the study because their scores were sharply (−3 SDs) lower than other students'. In addition, four other students withdrew from the study after the first pre-tests due to personal reasons. Therefore, a total of 34 participants remained active until the end. The participants were in three intact classes. The first class contained 12 students, the second 9, and the third 13. The rationale behind selecting upper-intermediate students was that they were supposed to possess satisfactory English proficiency to comprehend the language in audio-visual materials effectively. Prior to the administration of the placement test, informed consent was given to the samples following the APA ethical guidelines. The descriptive statistics of the participants are shown in Table 1.

**Table 1:** The descriptive statistics of the participants by group, gender, and age.

		**Gender**			**Age**			
	**Male**	**Female**	**Total**	**Min**	**Max**	**Mean**	**SD**	
Group	1	8 (66.7%)	4 (33.3%)	12	20	40	27.33	5.10
	2	4 (44.4%)	5 (55.6%)	9	20	35	28	4.66
	3	6 (46.2%)	7 (53.8%)	13	23	36	27.61	4.13
Total	18 (52.9%)	16 (47.1%)	34	20	40	27.61	4.49	

### Instruments

The following instruments were used to gather the required data.

#### Oxford Quick Placement Test

The placement test used for the study was the 60-item OQPT (2001). This test is devised by Oxford University Press (OUP) and the University of Cambridge Local Examinations Syndicate (UCLES) (Syndicate et al., 2001). It is divided into two parts. Parts one and two contain questions 1–40 and 41–60, respectively. This test is reported to be standardized, and thus its validity, reliability, and item difficulty are at a satisfactory level. The score-level criteria (Afshinfar and Shokouhifar, 2016) are presented in Appendix A.

#### Intercultural Sensitivity Scale

A 5-point 24-item Likert scale (form 1 = *strongly disagree* to 5 = *strongly agree*), by Chen and Starosta (2000) was administered to the participants. The intercultural sensitivity scale (ISS) (Appendix B) comprises of five factors, namely interaction engagement, respect for cultural differences, interaction confidence, interaction enjoyment, and interaction attentiveness. The reliability of this scale was reported to be 0.86.

#### Intercultural Effectiveness Scale

Related data were collected through the intercultural effectiveness scale (IES) (Portalla and Chen, 2010), a 20-item 5-point Likert scale. IES (Appendix C) is classified into six subscales, namely, behavioral flexibility, interaction relaxation, interactant respect, message skills, identity maintenance, and interaction management. The reliability of this instrument was reported to be 0.85.

#### Perception Semi-structured Interview

Considering the participants' perceptions toward the application of FC, a semi-structured interview was used (see Appendix D). Face-to-face interviews were conducted with the three groups, each including six individuals to assess the perception of participants toward the FC. Various questions were carefully developed as a guide for the interviewer to help contextualize and elicit the required data. The questions were reviewed by one researcher experienced in qualitative research methodology and teaching English to provide feedback concerning the accuracy and suitability of the items to obtain the related information. Yes/no and open-ended questions were utilized to allow for a deeper understanding of the phenomenon under study.

### Procedure

Having filled an informed consent form, non-randomly selected participants took the OQPT (30–35 min) and completed the English versions of IES and ISS. The participants were then presented with flipped cultural contents (see Appendix E) through the *Friends* American sitcom, uploaded to an accompanying website (http://www.englishpaths.ir). Participants were required to watch them and answer a follow-up quiz (see Appendix F). There were online discussion meetings the next day, and the researcher discussed cultural questions carefully tailored to the aims of IES and ISS (see Appendix G). The sessions were held twice a week using the Adobe Connect application. A total of 20 selected episodes were chosen, and the intervention lasted 10 weeks. At the end of week 10, a re-conduction of ICS and IES was performed to analyze how the intervention had affected participants' ICS and ICE. The data collection was finalized by holding two semi-structured interview sessions, recording them, and analyzing the data. The presentation of the audio-visual materials was administered in two phases for each episode. Detailed descriptions of the phases are presented below.

#### Phase 1

Webb and Nation (2018) introduced four strands for teaching vocabulary, namely meaning-focused input, meaning-focused output, language-focused learning, and fluency development, the first two of which target the receptive and productive skills, respectively. Drawing on Webb and Nation's (2018) and Brown's (2010) research, the researchers presented some e-vocabulary (Appendix H) mined from the episode to-be-watched to activate the participants' cultural schemata (Mahmoudi, 2017). This approach is also in line with Roell's (2010) pre-viewing activities. The participants then were to watch the assigned episode and answer some related e-questions to make sure they had watched it to the end. They subconsciously followed the while-viewing activities proposed by Roell (2010). The participants were to keep a post-viewing summary of any cultural peculiarities they found in the corresponding episode (Brown, 2010; Roell, 2010; Argynbayev et al., 2014).

#### Phase 2

The day after, the researcher held three 90-min sessions for the groups under study whose first activity was for one of the participants to summarize the episode in short orally (Brown, 2010). Next, the teacher-researcher posed some Y/N and Wh-Qs constructed carefully according to Vygotsky's SCT, Deardorff's process model, Byram's (1997) Savoirs, Bennett and Bennett's (2004) spectrum, and Lave and Wenger's (1991) situated learning. The researchers carefully designed the discussion questions based on ISS and IES constructs (Appendix G). At the end of the study, a semi-structured interview was presented to 22 participants, randomly selected from 3 classes. The interviews were conducted in Persian. The data were recorded, and the content analysis was performed *via* MAXQDA 2020. The qualitative data in Phase 2 of the study were analyzed through narrative content analysis. The first author along with a qualified research assistant transcribed the audio-taped files and the discussion notes in a Microsoft Word file. All the transcriptions were translated into English. The translated data were transferred to MAXQDA 2020 software. Following McCrudden and Barnes (2016), a thematic analysis was launched in a five-step process. First, the two researchers read and re-read the manuscripts independently to familiarize themselves with the data. Second, both researchers compared each participant's qualitative data with his/her quantitative data (pre-test and post-test). Third, initial codes were generated. In the fourth step, the researchers generated categories by aggregating similar codes. In the fifth step, the themes were identified, and the relevant relationships according to participants' responses were compared and examined. The first and second researchers finally discussed the emerging codes and themes to ensure inter-rater agreement. Any disagreements or disconfirming evidence were reanalyzed and resolved. Inference quality, reliability, validity, credibility, transferability, dependability, and confirmability assumptions were closely met throughout the study. The results are reported below.

## Results

In what follows the quantitative results followed by the qualitative results are presented.

### The Effect of Audio-Visuals on the Enhancement of ICS and ICE

The descriptive statistics of the variables are shown in Table 2.

**Table 2:** Descriptive statistics of the variables.

	***N***	**Min**	**Max**	**Mean**	**SE Mean**	**SD**	**Variance**	**Cronbach's α**
ICS Pre-test	34	70.00	97.00	85.67	1.20	7.00	49.13	0.84
ICE Pre-test	34	63.00	80.00	71.41	.90	5.29	28.00	0.81
ICS Post-test	34	82.00	111.00	98.08	1.34	7.86	61.84	0.82
ICE Post-test	34	72.00	91.00	83.05	.89	5.23	27.45	0.85
OQPT	34	47.00	56.00	51.23	.50	2.93	8.61	0.92

*ICS, intercultural sensitivity; ICE, intercultural effectiveness; OQPT, Oxford Quick Placement Test*.

To answer the RQ1 and RQ2 of the study, two-paired sample *t*-test analyses were run (Table 3). The assumptions of normality and homogeneity of variance were explored before conducting the main statistical analyses. The results did not show any deviations from the presumed assumptions.

**Table 3:** Paired-samples *t*-test analyses of ICS and ICE.

		**Mean**	***N***	**SE Mean**	***R***	***t***	**df**	**Sig**.
Pair 1	ICS Pre-test	85.67	34	0.95	0.727	−13.02	33	0.000
	ICS Post-test	98.08	34					
Pair 2	ICE Pre-test	71.41	34	0.82	0.581	−14.08	33	0.000
	ICE Post-test	83.05	34					

Considering the first research question, the participants obtained significantly higher scores on ICS post-test (M = 83.05, SD = 5.23, SE = 0.89) compared to their pre-test scores on the ICS (M = 71.41, SD = 5.29, SE = 0.90), *t* (33) = −13.02, *p* = 0.000, *r* = 0.727.

Regarding the second research question, the participants obtained significantly higher scores on ICE post-test (M = 98.08, SD = 7.86, SE = 1.34) compared to their pre-test scores on the ICE (M = 85.67, SD = 7, SE = 1.20), *t* (33) = −13.02, *p* = 0.000, *r* = 0.727.

In sum, the results indicate that the treatment had a significant effect on participants' intercultural sensitivity and effectiveness. Though, intercultural sensitivity scores proved higher improvement. The qualitative data were also recorded; however, reporting the qualitative data is not within the scope of this study.

### The Perception of the Participants on FC

Regarding the third research questions, which investigated the perception and attitudes of the participants on FC and after a content analysis, several themes emerged. The data are depicted in Table 4.

**Table 4:** General themes developed from semi-structured interviews.

**Attitudes**	**General codes**	**Emerged themes**	**Students' example responses**
The students' attitudes toward the use of flipped classroom	Flexible environment	Mode of watching videos: online	I could watch *Friends via* internet freely.
		The time of watching videos: 24/7	I am usually at work till 9 p.m. and could watch them whenever I am free.
		Times of watching videos: unlimited	I usually watched each episode 3 times during a day.
		Medium of watching videos: any electronic device	It was really good that I could watch the series on my mobile, laptop or smart TV.
		The place of watching videos: anywhere	I had to travel to Tehran for 1 week and I watched *Friends* there.
	Learning culture	Watching videos prior to the class: activating schemata; feeling confident	It was perfect that we had the opportunity to watch videos before the class. That is why I personally felt really confident in discussions.
		Class time allocation: exploring topics in great depth	I felt that unlike some other classes, the class was very active and we did not waste time. We had a lot of speaking.
	Using videos	Being immersed: authentic language	I felt the English culture and beliefs while watching *Friends*. It was better than reading books.
		Genuineness of videos: everyday life	Previously I thought the English lived a different everyday life. You know *Friends* helped me understand what they really did each day.
		Timing of videos: short episodes	I think because the length of videos was short, I watched them all (chuckling).
		Outlook of videos: appealing situations	OMG, it was one of the best series I had ever watched. I enjoyed every second of it.
		Peripheral impact of videos: critical thinking about the situations	When I spoke to my peers in break-out rooms, we discussed if we could really judge the English as having a good culture or not.
The students' attitudes toward the use of technology	Using website	Ease of use: time and location of use	The website you used was very handy and user-friendly. I could use it whenever and wherever I wanted.
		Self-regulated learning: times of watching	I thought watching videos on the website was very good. It was like YouTube. I watched some parts many times
	Using Adobe connect	Break-out rooms: more interaction	Sometimes I didn't want to leave the rooms. You know I love speaking with others. The topics were also so interesting.
		Easiness: from any place; any time	As I said before, I traveled to Tehran and attended the class from there. There was one session I could not attend. But Peyman recorded the class for me.
		Less face-threatening: freedom of speech	To be honest I am shy. Sometimes I didn't say my idea in the class for some reasons. But I discussed my opinions in break-out rooms with my friends openly.

The semi-structured interview questions (Appendix F) were used to explore the participants' attitudes toward FC. However, after the content analysis, the researchers, as it can be seen in Table 4, came up with the attitudes of the participants about the use of technology. There came to be four general codes for the students' attitudes toward the use of FC and 2 general codes for the students' attitudes toward the use of technology. Moreover, as the qualitative research may be exploratory, according to the answers of the participants, 17 themes emerged for opinions on FC and on technology.

## Discussion and Conclusions

This study attempted to investigate the effect of culture-embedded audio-visual *Friends* sitcom on ICS and ICE of EFL students in English language institutes in Kerman. After the two pre- and post-administrations of ISS and IES, the results revealed that both ICS and ICE improved. Though, the samples indicated more improvement in ICS. This enhancement is presumed to be the effect of the intervention.

Regarding the positive effect of FC and authentic audio-visual materials on the enhancement of intercultural competence in general and intercultural sensitivity and effectiveness in particular, the results of this study confirm the findings of Desai et al. (2018), Zhang (2020), and Jensen (2019). Consequently, the development of both ICS and ICE in this study following the models of Byram (1997), Deardorff (2006), and Bennett (2004) reveals that intercultural development through culture-simulated audio-visual authentic material accompanied by scaffolding discussions is plausible in an EFL context where direct exposure to the foreign culture seems improbable (Namaziandost et al., 2022).

Unlike the first research question, to the best knowledge of the researchers, no data were available to correlate the findings of the second research question to the previous studies. Consequently, further research should examine this issue and unravel its unresolved sides. However, the overall findings confirm the positive impact of incorporating culture into the curriculum.

Considering the third research question, the data show positive attitudes of the participants about using FC, which confirms the findings of some other previous research (Galway et al., 2015; Zainuddin and Attaran, 2016). However, the findings of He et al. (2016) showed contradicting attitudes according to which some individuals had positive views, which support the findings of this research, though some others had opposite views compared to the current study.

The findings of this study provide some practical implications for language teachers and students. Since EFL students do not have enough exposure to FL outside the classroom (Ziashahabi et al., 2020; Jamali et al., 2021), this research suggests that students get involved in L2 and its indispensably entwined cultural aspects by flipping the materials. Moreover, FC will enhance students' autonomy and self-regulation by offering 24/7 learning opportunities and consequently foster life-long learning. Besides, through MKO and ZPD, collaboration and schemata activation will be emphasized. Therefore, flipping classes will greatly enhance EFL students' cultural understanding.

As mentioned before, the positive perceptions of individuals can be reflective of their intrinsic motivation. Therefore, if individuals develop positivity on the perception continuum, it will lead to better educational achievement. The findings of this study showed positive attitudes toward FC and the use of technology. Consequently, this mutual relationship of positive perception and motivation may give rise to the further achievement of students. This study, hence, encourages the use of FC and technology in educational settings.

The findings of the study should be interpreted in the context of some limitations. This research studied the effect of FC on ICS and ICE in the EFL context of Iran, yet the findings are not generalizable worldwide as other EFL contexts might have diverse social and cultural textures. Another issue is that all the participants of this study were would-be immigrants; therefore, the results may not account for non-immigrants. Consequently, the mentioned limitations call for the replications of this study in other EFL contexts and with larger samples.

This study used individuals in the source country where they could not integrate (Bennett, 2004), manifest external outcomes (Deardorff, 2004, 2006), and fulfill Savoirs' engager (Byram, 1997). Consequently, the ultimate goal of languaculture (Lantolf, 2007) was ignored. The researchers suggest other scholars explore whether any source country samples would perform in the target country culturally effectively and appropriately so as to see if the models of intercultural competencies are completed thoroughly in target countries.

## Data Availability Statement

The original contributions presented in the study are included in the article/Supplementary Material, further inquiries can be directed to the corresponding author/s.

## Ethics Statement

The studies involving human participants were reviewed and approved by Yazd University. The participants provided their written informed consent to participate in this study.

## Author Contributions

All authors listed have made a substantial, direct, and intellectual contribution to the work and approved it for publication.

## Conflict of Interest

The authors declare that the research was conducted in the absence of any commercial or financial relationships that could be construed as a potential conflict of interest.

## Publisher's Note

All claims expressed in this article are solely those of the authors and do not necessarily represent those of their affiliated organizations, or those of the publisher, the editors and the reviewers. Any product that may be evaluated in this article, or claim that may be made by its manufacturer, is not guaranteed or endorsed by the publisher.
