# Supplementary material for: Flipped Presentation of Authentic Audio-Visual Materials: Impacts on Intercultural Sensitivity and Intercultural Effectiveness in an EFL Context
Source: Front Psychol. 2022 Feb 21;13:832862. doi: 10.3389/fpsyg.2022.832862 (PMC8899533; doi:10.3389/fpsyg.2022.832862)
Supplement: Supplementary file 1 [file Table_1.pdf]

Appendix A.

The score level criteria for OQPT

Score	Level
0–16	A1- Elementary
17–27	A2 - Pre-intermediate
28–36	B1 - Intermediate
37–44	B2 - Upper-intermediate
45–54	C1 - Advanced
55–60	C2 - Proficient

Appendix B.

Intercultural sensitivity scale

Direction: Below is a series of statements concerning intercultural communication. There are no right or wrong answers. Please work quickly and record your first impression by indicating the degree to which you agree or disagree with the statement. Thank you for your cooperation.

5 = strongly agree<br>4 = agree<br>3 = uncertain<br>2 = disagree<br>1 = strongly disagree	Please put the number corresponding to your answer in the blank before the statement

____ 1. I enjoy interacting with people from different cultures.
____ 2. I think people from other cultures are narrow-minded.
____ 3. I am pretty sure of myself in interacting with people from different cultures.
____ 4. I find it very hard to talk in front of people from different cultures.
____ 5. I always know what to say when interacting with people from different cultures.
____ 6. I can be as sociable as I want to be when interacting with people from different cultures.
____ 7. I don't like 10 be with people from different cultures.
____ 8. I respect the values of people from different cultures.
____ 9. I get upset easily when interacting with people from different cultures.
____ 10. I feel confident when interacting with people from different cultures.
____ 11. I tend to wait before forming an impression of culturally distinct counterparts.
____ 12. I often gel discouraged when I am with people from different cultures.
____ 13. I am open-minded to people from different cultures.
____ 14. I am very observant when interacting with people from different cultures.
____ 15. I often feel useless when interacting with people from different cultures.
____ 16. I respect the ways people from different cultures behave.
____ 17. I try to obtain as much information as I can when interacting with people from different cultures.
____ 18. I would not accept the opinions of people from different cultures.
____ 19. I am sensitive to my culturally distinct counterpart's subtle meanings during our interaction.
____ 20. I think my culture is better than other cultures.

____ 21. I often give positive responses to my culturally different counterpart during our interaction.
____ 22. I avoid those situations where I will have to deal with culturally distinct persons.
____ 23. I often show my culturally distinct counterpart my understanding through verbal or nonverbal cues.
____ 24. I have a feeling of enjoyment toward differences between my culturally distinct counterpart and me.

Note. Items 2, 4, 7, 9, 12, 15, 18, 20, and 22 are reverse-coded before summing the 24 items. Interaction Engagement items are 1, 11, 13, 21, 22, 23, and 24, Respect for Cultural Differences items are 2, 7, 8, 16, 18, and 20, Interaction Confidence items are 3, 4, 5, 6, and 10, Interaction Enjoyment items are 9, 12, and 15, and Interaction Attentiveness items arc 14, 17, and 19.

Appendix C.

Intercultural Effectiveness Scale

Direction: Below is a series of statements concerning intercultural communication. There are no right or wrong answers. Please work quickly and record your first impression by indicating the degree to which you agree or disagree with the statement. Thank you for your cooperation.

5 = strongly agree<br>4 = agree<br>3 = uncertain<br>2 = disagree<br>1 = strongly disagree	Please put the number corresponding to your answer in the blank before the statement

____ 1. I find it is easy to talk with people from different cultures.
____ 2. I am afraid to express myself when interacting with people from different cultures.
____ 3. I find it is easy to get along with people from different cultures.
____ 4. I am not always the person I appear to be when interacting with people from different cultures.
____ 5. I am able to express my ideas clearly when interacting with people from different cultures.
____ 6. I have problems with grammar when interacting with people from different cultures.
____ 7. I am able to answer questions effectively when interacting with people from different cultures.
____ 8. I find it is difficult to feel my culturally different counterparts are similar to me.
____ 9. I use appropriate eye contact when interacting with people from different cultures.
____ 10. I have problems distinguishing between informative and persuasive messages when interacting with people from different cultures.
____ 11. I always know how to initiate a conversation when interacting with people from different cultures.
____ 12. I often miss parts of what is going on when interacting with people from different cultures.
____ 13. I feel relaxed when interacting with people from different cultures.
____ 14. I often act like a very different person when interacting with people from different cultures.
____ 15. I always show respect for my culturally different counterparts during our interaction.
____ 16. I always feel a sense of distance with my culturally different counterparts during our interaction.
____ 17. I find I have a lot in common with my culturally different counterparts during our interaction.
____ 18. I find the best way to act is to be myself when interacting with people from different cultures.
____ 19. I find it is easy to identify with my culturally different counterparts during our interaction.
____ 20. I always show respect for the opinions of my culturally different counterparts during our interaction.

Note. Items 2, 4, 6, 8, 10, 12, 14, 16, and 18 are reverse-coded before summing the 20 items. Behavioral Flexibility items are 2, 4, 14, and 18; Interaction Relaxation items are 1, 3, 11, 13, and 19; Interactant Respect items are 9, 15, and 20; Message Skills items are 6, 10, and 12; Identity Maintenance items are 8, 16, and 17; Interaction Management items are 5 and 7.

Appendix D.

Semi-structured interview questions

	Questions	Aspect
1	How was your experience of being in this class?	General
2	What aspects of the class did you like?	General
3	How was your experience of watching videos? Explain to me.	Videos
4	Do you think it is beneficial to watch videos before coming to class?	Timing
5	What are some of benefits of watching videos before the class time?	Videos + Timing
6	Was it easy to play videos? Could you watch them anywhere/anytime?	Flexible environment
7	What is your idea about discussions in the class?	Dialogic Interaction
8	Do you think that the teacher was helpful during the class?	Professional educator
9	How did you feel about spending time in class?	Learning culture
10	Do you think the videos were related to the discussion	Intentional content
11	Please tell me about break-out rooms? Did you like them?	Team-work

Appendix E.

Friends seasons, episodes, and cultural themes

#	Season & Episode (N.N)	Cultural Theme
1	1.1/ 4.24/5.24/7.23	Marriage and Wedding Ceremony
2	6.1	Divorce
3	Prevalent	Friendship Before Marriage
4	Prevalent	Independence age

5	Prevalent	Living separate from Parents
6	Prevalent	Drinking Alcohol
7	1.1/1.2/1.23/1.9	Sexuality: Homosexual, Lesbian
8	Prevalent	Friends Living together
9	1.10	Christmas
10	1.9	Thanksgiving
11	Prevalent	Friends or Family: The counselor
12	Prevalent	Taboo in speech
13	1.18	Male–Female discrimination
14	Prevalent	Freedom of speech: respect to others (minority group: The Black)

Appendix F.

A sample follow-up quiz

#	**Questions**	**Expected answers***
1	Where did Marcell come from?	Ross's friend rescued him from a lab
2	What did the group agree to do on New Year's Eve?	Not to bring dates/ just have dinner together
3	Who is David?	Phoebe met him at the cafe and they began to date
4	Who did Phoebe/Chandler/Joey/Monica invite?	David/Janice/Cindy/fun Bobby
5	Where was David going?	Minsk
6	What happened the last time Marcell was in Monica's house?	He urinated on her coffee table.
7	What happened to Rachel on the way to Monica's house?	She got into a fight
8	What happened to fun Bobby?	His grandfather died
9	What does David decide to do at last?	Go to Minsk

10	What happened to the group's plan for New Year's Eve?	They end up without dates

Appendix G

Discussion Questions according to the theoretical framework drawing on Deardorff's Process Model, Byram's Model, and Bennet's Model

1. Did you experience culture shock when watching the film? (Attitude) (Savoir être)
2. Do you reject this behavior? (denial)
3. What behaviors did you find strange and weird? (Attitude) (Savoir être)
4. Do you think our culture handles this issue better? (Defense)
5. What cultural issues did you see that you are curious about to know more? (Attitude: curiosity)
6. Do you think this behavior is normal? If no, why then did the characters do it? (Attitude: openness)
7. What cultural behaviors/issues did you see in the episode? (Observation skill)
8. What do you think of those behavior/issues? (Evaluating and analyzing skill)
9. What was the purpose of that behavior/issue? (Interpreting skill) (Savoir comprendre)
10. What is a normal reaction to this situation in your country? (Relating skill) (Savoir comprendre)
11. Do you think this behavior is fine? How can your cultural beliefs accept this? (Reversal)
12. Regarding this behavior, do you think the English culture faces it better or you cultures does better? (Reversal)
13. How would the English judge your reaction in this situation? What about other cultures? (Knowledge) (Savoirs)
14. Does this behavior have the same meaning/connotation in different cultures? (Global Knowledge) (Savoirs)
15. Do you think you can accept these issues? (Internal outcome: flexibility and Adaptability)
16. Do you think these issues could be normal regardless of what you have in your culture? (Internal outcome: ethnorelative perspective)
17. Do you think these differences are because of cultural misunderstandings? (Savoir apprendre)
18. Regarding the English culture, do you think that the English will accept these issues openly? (Internal outcome: empathy)
19. Do you think it is ok to show this behavior in an English culture? (Internal outcome: empathy)
20. Do you think that this behavior can be something normal and other cultures may accept it? (Minimization)
21. Do you think that this issue should be culture-bound or it should be considered regardless of any cultures? (Minimization)

22. If you were in an English culture, do you think that you would have the same behavior? (External outcome: Effectiveness) (Adaptation)
23. Imagine that now you are in an English culture and have accepted it, will you do the same behavior? (Integration)
24. Do you think this behavior would be judged as appropriate buy others? (External outcome: Appropriateness)
25. Do you think you cannot accept this behavior just because we are Iranians ad have a different culture? (Savoirs' engager)

Appendix H.

Vocabulary for Episode about Christmas (season 1, episode 10)

1. Christmas

2. Snowman

3. Santa

4. New Year

5. Holiday

6. Christmas tree

7. New Year's Eve

8. Midnight ball drop
